# Magnetic field distribution modulation of intrathecal delivered ketorolac iron-oxide nanoparticle conjugates produce excellent analgesia for chronic inflammatory pain

**DOI:** 10.1186/s12951-018-0375-9

**Published:** 2018-05-16

**Authors:** Ping-Ching Wu, Dar-Bin Shieh, Hung-Tsung Hsiao, Jeffery Chi-Fei Wang, Ya-Chi Lin, Yen-Chin Liu

**Affiliations:** 1 Department of Biomedical Engineering, College of Engineering, National Cheng Kung University, Tainan, Taiwan; 2 Institute of Basic Medical Science, National Cheng Kung University, Tainan, Taiwan; 3 Institute of Oral Medicine, National Cheng Kung University, Tainan, Taiwan; 4 Department of Stomatology, Institute of Basic Medical Science, College of Medicine, National Cheng Kung University, Tainan, Taiwan; 5 Medical Device Innovation Center, Taiwan Innovation Center of Medical Devices and Technology, National Cheng Kung University Hospital, National Cheng Kung University, Tainan, Taiwan; 6 Advanced Optoelectronic Technology Center, National Cheng Kung University, Tainan, Taiwan; 7 Center for Micro/Nano Science and Technology, National Cheng Kung University, Tainan, Taiwan; 8 Department of Anesthesiology, National Cheng Kung University Hospital, College of Medicine, National Cheng Kung University, Tainan, Taiwan

**Keywords:** Ketorolac, Ultra small iron nanoparticles, Inflammatory pain, Cyclooxygenase, Magnetic field

## Abstract

**Background:**

Nanoparticles have become one of the most promising among the potential materials used for biomedical applications. However, few researchers have focused on their effects on analgesia. Despite the fact that various nanoparticles have been evaluated for drug delivery and MRI imaging contrast enhancement in clinical settings, no reports have investigated the in vivo synergy of ketorolac iron-oxide nanoparticle conjugates to improve the analgesic effect.

**Methods:**

Ketorolac conjugated magnetic iron oxide nanoparticles (Keto-SPIO) were synthesized via two-stage additions of protective agents and chemical co-precipitation. ICR mice were used to develop inflammatory pain models induced by Complete Freund’s adjuvant (CFA) injection in the hind paw. Different magnet field strengths and polarities were applied to the spinal cord after injecting Keto-SPIO into the theca space. Analgesia behavior was evaluated with the up-down method via von Frey microfilament measurement. Spinal cord tissues were harvested at the end analgesia time point upon induction of the inflammatory pain. The presence of the two cyclooxygenases (COX) in the spinal cord was examined via Western blotting to quantify the changes after intra-thecal Keto-SPIO administration.

**Results:**

Intrathecal Keto-SPIO administration demonstrated a magnetic field-dependent analgesia effect in CFA pain model with a significant reduction in COX expression.

**Conclusions:**

Our results indicated that intrathecal administration of the Keto-SPIO combined magnet field modulated delivery significantly promoted an analgesia effect with suppression of COX in the mice inflammatory pain model.

**Electronic supplementary material:**

The online version of this article (10.1186/s12951-018-0375-9) contains supplementary material, which is available to authorized users.

## Background

Magnetic nanoparticles (NP) have been used as vectors to deliver drugs to specific target sites. Superparamagnetic iron oxide nanoparticles have been engineered to enhance tumor magnetic resonance imaging (MRI) contrast based on their differential uptake by brain parenchymal cells and their effect on the microenvironment magnetic field [1]. Superparamagnetic iron oxide nanoparticles (SPIO) have also been applied to enable monitoring of not only anatomical changes but also functional and molecular level alterations of diseases under MRI as well [2]. Koji et al. demonstrated minimally invasive transplantation of bone marrow stromal cells (BMSCs) via the lumbar puncture followed by magnetic targeting of BMSCs to the desired site in the spinal cord through cerebrospinal fluid route delivery [3]. Their application in magnetic field guided analgesia has, however, not been reported to date.

Non-steroid anti-inflammatory drugs (NSAIDs) are one of the most prescribed drugs in the world for pain relief and control of inflammation and fever via inhibiting prostaglandins (PGs) synthesis [4]. Intrathecal administration of ketorolac has been approved for clinical analgesia [5]. We recently demonstrated the local analgesic effect of SPIOs in an in vivo inflammatory pain model [6]. It is thus conceivable that the integration of magnetic field-guided delivery via the superparamagnetic property of SPIOs and their endogenous analgesic activity with co-delivery of effective NSAIDS may have synergistic efficacy. Here, we demonstrated the validity of this idea using SPIO nanoparticles conjugated with ketorolac as a novel intrathecal route analgesic combination and presented excellent outcome in the mouse chronic inflammatory pain model through magnetic field-guided delivery with defined magnetic polarity.

## Methods

### Ketorolac iron-oxide nanoparticle conjugate (Keto-SPIO) preparation

#### Iron nanoparticle preparation

To produce the Fe_3_O_4_ nanoparticles, 1 ml of Fe(II) and 4 ml of Fe(III) aqueous solutions were mixed at room temperature, followed by the addition of 0.5 g (25%)(w/w) tetramethylazanium hydroxide as an adherent. Added the 0.5 M NaOH just for adjust PH, the added volume was depended the reaction finished when the pH of the solution reached to 11 [7]. The nanoparticle size was determined to be about 6.2 ± 2.1 nm using transmission electron microscope. The amide terminated SPIOs were then synthesized for the next step.

#### Ketorolac iron-oxide nanoparticle conjugates (Keto-SPIO) synthesis

1.0 ml SPIO (Fe_3_O_4_, 10 mg/ml) was then reacted with 0.08 ml (30 mg/ml) ketorolac (042412, Yungshin Co. Taichung City, Taiwan). We added 0.08 ml ketorolac (30 mg/ml) into 1.0 ml of SPIO (Fe_3_O_4_, 10 mg/ml) nanoparticles plus 1-ethyl-3-(3-dimethylaminopropyl) carbodiimide (EDC, as a dehydration catalyst) to react for 8 h at room temperature. After 8 h, the liquid was then centrifugated at 12,000 rpm for 15 min. The supernatant was measured to identify the concentration of ketorolac and the optical density (O.D) (381 nm) was used to calculate the amount of ketorolac in the supernatant. The amount of ketorolac in the Keto-SPIO was then calculated after synthesis. The synthesized Keto-SPIO was then dissolved in 1000 μl of phosphate-buffered saline (PBS) before intrathecal administration. Finally, the amount of keto-SPIO was 0.2352 mg/mg were dissolved in 1000 µl of PBS and the conjugated ketorolac should have a 0.117 absorbance calculated as 1.17 mg in the Keto-SPIO.

### Distribution tracking SPIO after intrathecal administration

We used the Xenogen IVIS^®^ Spectrum Noninvasive Quantitative Molecular Imaging System (PerkinElmer Inc. Waltham, MA) to real-time tract the distribution of the SPIO conjugates after injection into the spinal cords of the mice. For IVIS^®^, the Indocyanine green (ICG) (negatively charged sulfate group) (Sigma Aldrich, St. Louis, MO) labeled SPIOs (positive surface charge) [7] were reproduced by conjugating 10 μl (66.8 μM) of SPIO to 0.0009 g of ICG in 1000 μl of deionized water, which were then reacted for 2 h. After centrifugated at 12,000 rpm for 15 min to removed non-conjugate ICG, IGC conjugated SPIOs (ICG-SPIO) then wash phosphate-buffered saline (PBS) and repeat three times. The final products were obtained and washed via several cycles of dispersion in 500 μl PBS. ICG-SPIO were synthesized and intrathecally administrated into the spinal cords of the mice (10 μl/mice). The mice were anesthetized lightly (1.5–2.5% isoflurane) to keep them stationary during the injection, as previously described [8, 9]. After the ICG-SPIO were injected, a neodymium magnet [15 mm (diameter)*3 mm (thickness), Leap Tong Ind. Co., Taiwan] with a surface magnetic field about 2518 ± 10% gauss was placed in the injected area of the mice lumbar spine for 30 min. Different magnet polarity (N-S: attracting pole, N-N: repel pole, distance: 15 mm) were also tested to check to the influence of the magnet field on the efficacy. The real-time bioluminescent imaging was performed using IVIS^®^ imaging system for a total 3 h. Mice were repetitively scanned over a time period of 0–180 min, while body temperature was kept on a 37 °C platform (default setting for fluorescence imaging of ICG: excitation wavelength 745 nm, emission 840 nm, automatic exposure). All bioluminescent imaging were formatted with the same color coded scale for visual assessment. Bioluminescence from the region (ROIs) of interest was manually delineated, and the data were expressed as photon-flux (photons/s/cm^2^/steradian). Background photon-flux was defined using an ROI that was after intrathecal administration (0 min). The bioluminescent data were collected and analyzed using IVIS^®^ Living image system.

### Animal pain model and behavioural analysis

Male CD1 mice (20–30 g) were used and all animal studies were approved by National Cheng Kung Medical College Animal Care Guidelines (IACUC Approval No: 100047). The behavior check were followed our previous study [6, 10] with Dixon’s up-down method [11]. The inflammatory pain condition was also induced as our previous Complete Freund’s Adjuvant (CFA) model [6, 10]. After inflammatory pain was established, all test groups [ketorolac 10 mg/ml, Keto-SPIO (N-N, N-S, no magnet) and SPIO (Fe_3_O_4_) 10 mg/ml (N-N, N-S)] received a lumbar intrathecal injection with 10 μl of the test solution under 1% isoflurane anesthesia. Then the neodymium magnet was placed at the lumbar spine level and kept there for 30 min under light anesthesia (1% isoflurane). Then, the anesthesia was stopped, and the mice were put back on the elevated metal mesh for recovery and further behavioral measurements. Different doses of ketorolac (10 mg/ml) and SPIO (10 mg/ml) were also used to evaluate the optimal chemical conjugation dose. The analgesic effect of the drugs was evaluated every 30 min up to 180 min. The mice were sacrificed under deep anesthesia (5% isoflurane) at the end of the von Frey hairs testing (180 min). The spinal cord (left dorsal lesion quarter only) were harvested and stored at − 80 °C before the examination.

### Protein expression

The tissue was homogenized in an SDS sample buffer containing a mixture of proteinase and phosphatase inhibitors (Sigma). The extracted protein (25 μg/μl) was separated on SDS–PAGE gels and transferred to nitrocellulose blots. The blots were blocked with 5% milk and incubated overnight at 4 °C with primary antibodies against cyclooxygenase-1 and 2 (COX-1, COX-2) (anti-COX-1 goat polyclonal antibody, 1:1000, cat# sc-1754, Santa Cruz Biotechnology Inc, Dallas, TX, USA and anti-COX-2 goat polyclonal antibody, 1:1000, cat# sc-1747, Santa Cruz Biotechnology Inc, Dallas, TX, USA). Endogenous β-actin was used as the loading control and was revealed using anti-human β-actin mouse monoclonal antibody (cat# MAB1501, EMD Millipore, Billerica, CA, USA). After incubation of the primary antibodies, the blots were washed and labeled with the respective secondary antibody, i.e. anti-mouse or anti-rabbit IgG (GE Healthcare Life Sciences) conjugated with horseradish peroxidase for 1 h at room temperature. Chemiluminescence detection was performed with an immobilon western chemiluminescent HRP substrate and measured directly using a BioSpectrum Imaging System (UVP). Specific bands were evaluated by apparent molecular size.

### Quantification and statistics

For data of IVIS, the maximum radiance of the photon intensity of all ROIs in each group was analyzed by one-way ANOVA for statistical comparison. Individual groups were compared by t test. For the behavioral studies, the data for the paw withdrawal thresholds passed the normality test and thus were suitable for the parametric statistics. The data were analyzed with either a Student’s t test (two groups only) or a one-way ANOVA followed by a Newman–Keuls test for the post hoc analysis. All the data were presented as mean ± SEM, and *p* < 0.05 was considered statistically significant.

For the quantification of the Western blot, the density of specific bands for COX-1, COX-2 and β-actin were measured with imaging analysis software (Image J, NIH). The size of the rectangle was fixed for each band and the background near that band was subtracted. The expression level of each protein was normalized to the loading controls (β-actin). All images were taken using identical illumination intensities and were analyzed using a computer-assisted imaging analysis system (Image J, NIH).

## Results

**Fig. 1:**
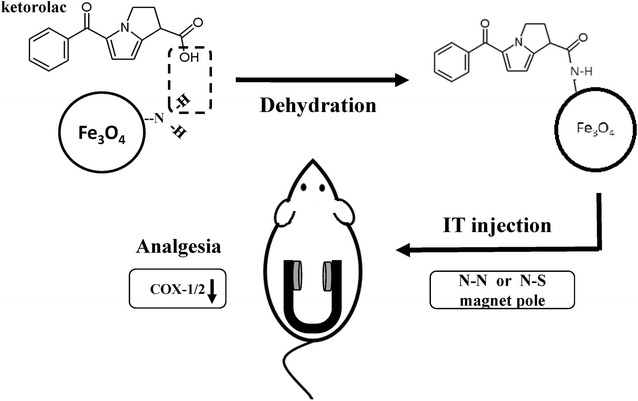
A brief summary of this research. The Ketorolac iron-oxide nanoparticle conjugate was synthesized and then injected into the mice spinal cords. Different magnet polarities were then applied to test the analgesic effect. The expression of COX-1 and COX-2 in the spinal cords of the mice were also checked

**Fig. 2:**
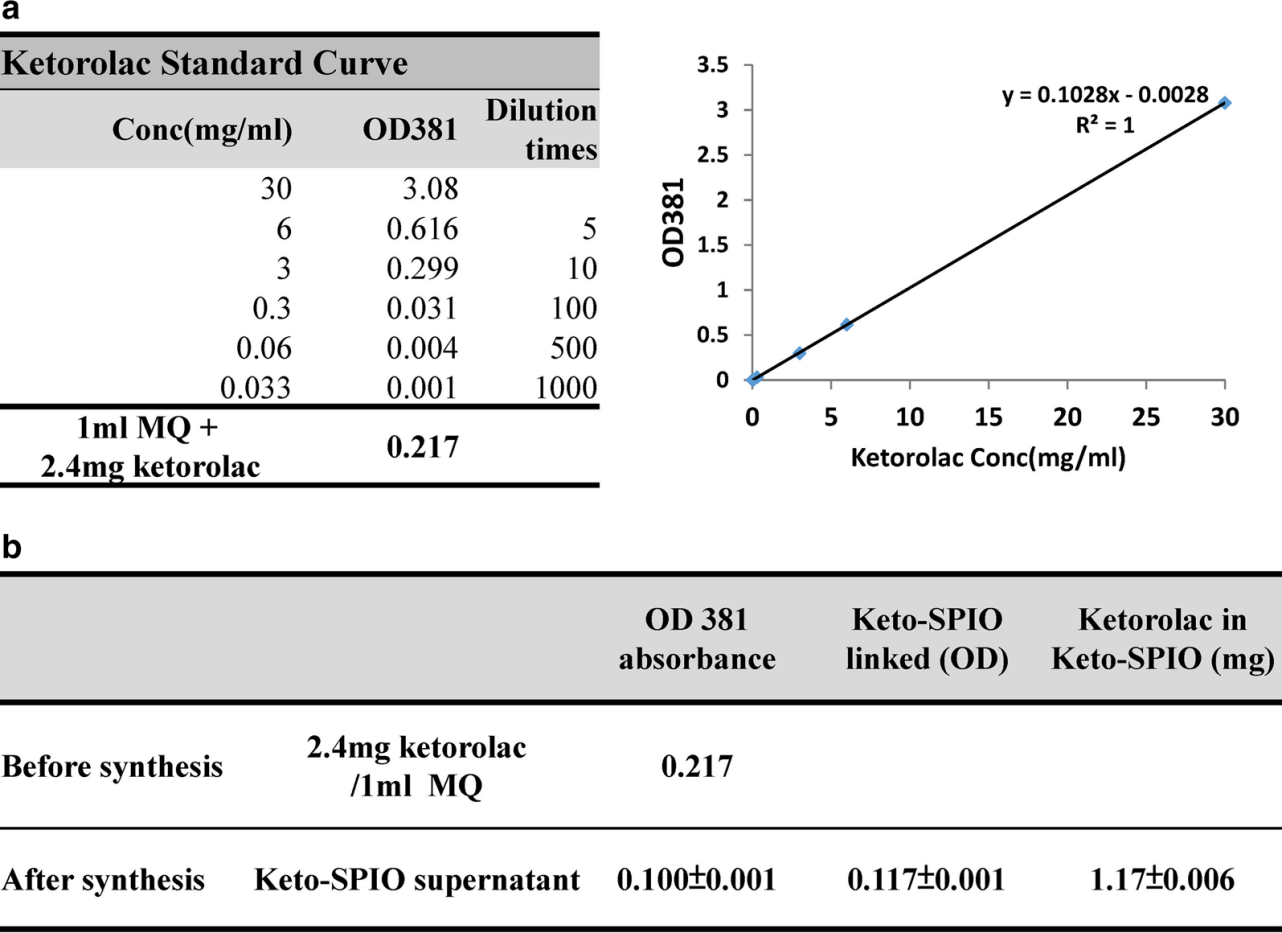
Demonstration of the calculated amount of Keto-SPIO injected into the mice spinal cord. Ketorolac exhibited good concentration-dependent absorbance on OD 381 nm (**a**). The amount of ketorolac and iron nanoparticles within the Keto-SPIO can be calculated via the substrate in the residual ketorolac in the supernatant after synthesis (**b**)

The concept of magnetic field guided analgesic nanomedicine delivery in this study is illustrated in Fig. 1. The ketorolac iron-oxide nanoparticle conjugate was synthesized via dehydration and then injected into the spinal cord of mice that were in pain. Different magnetic polarities were then applied to test the analgesic effect. The expression of COX-1 and COX-2 in the spinal cord was also checked. The amount of the ketorolac linked onto the Keto-SPIO NPs was calculated using optic absorbance (Fig. 2). A good correlation of ketorolac concentration and an optic absorbance of 381 nm was exhibited (Fig. 2a). The average amount of ketorolac conjugated on the SPIOs was also calculated via subtracting the residual ketorolac after synthesis (Fig. 2b). The loading capacity of the SPIO was 0.2352 mg/mg, and the conjugation efficiency was over 98%. The final analgesic injection amount was determined to be 11.75 μg ketorolac on 100 μg of SPIOs dissolved in 10 μl PBS per mouse per injection.

**Fig. 3:**
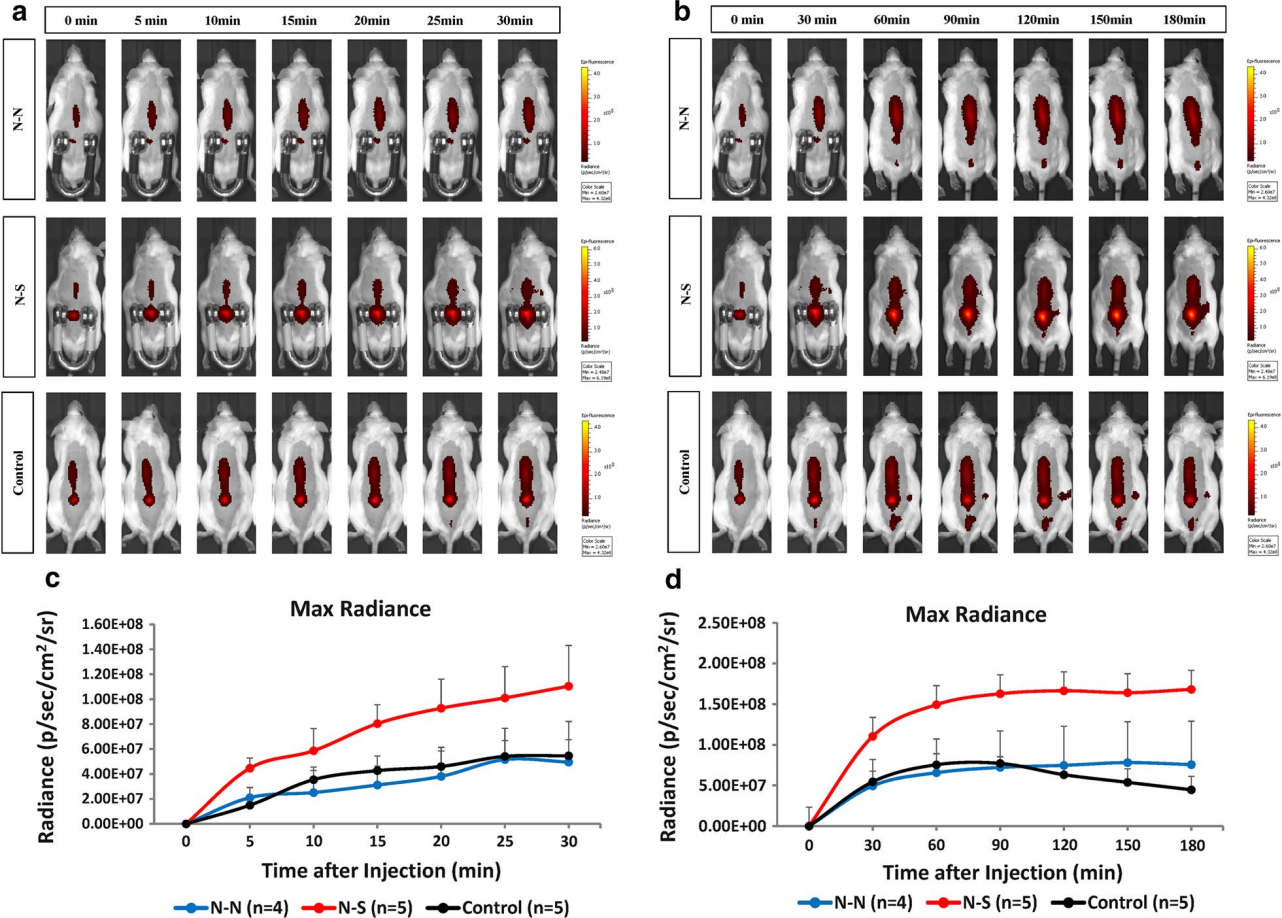
The live serial signal change in the iron nanoparticles (ICG-SPIO) after intrathecal administration [**a**, **c** (0–30 min, with magnet), **b**, **d** (0–180 min)]. The signal of different magnet polarities (N-S) seems to accumulate more in the lumbar area than in the same polarity (N-N) group. The bioluminescence intensity is indicated as photon-flux (photons/s/cm^2^/steradian). Values are mean ± S.E.M

The influence of the applied magnetic field to the local SPIO distribution within the spinal canal was assessed by image processing (Fig. 3). The SPIO signals presented in Fig. 3a, b revealed that the N-S magnetic field orientation arrangement presented a stronger but not significant fluorescent signal over the lumbar spine area than that of the N-N arrangement in maximum radiance (photons/s/cm^2^/steradian): (30 min) 1.10 × 10^8^ ± 3.25 × 10^7^ (N-S) vs. 4.96 × 10^7^ ± 2.89 × 10^7^ (N–N) and 5.46 × 10^7^ ± 1.30 × 10^7^ (control) (*p *= 0.22); [180 min] 1.68 × 10^8^ ± 5.34 × 10^7^ (N-S) vs. 7.57 × 10^7^ ± 1.38 × 10^7^ (N–N) and 4.47 × 10^7^ ± 1.64 × 10^7^ (control) (*p *= 0.07).

**Fig. 4:**
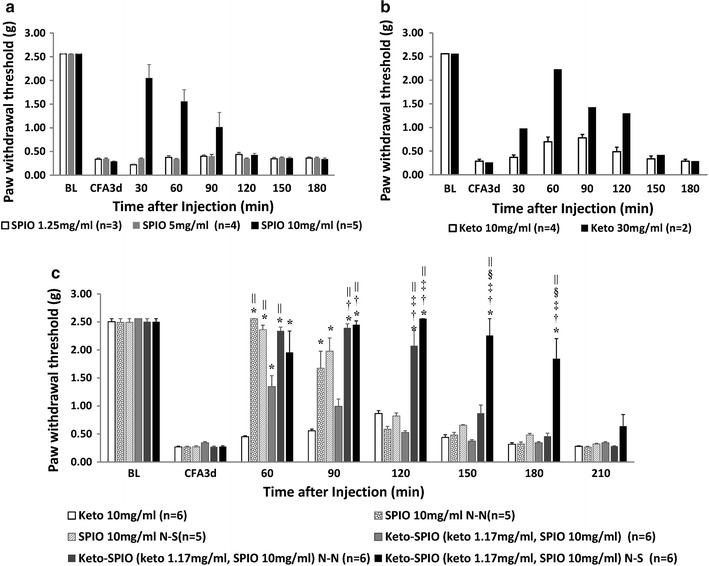
**a**, **b** demonstrate that 10 mg/ml of SPIO and 30 mg/ml of ketorolac can produce analgesic effects. The anti-allodynia effect of the Keto-SPIO on mice experiencing inflammatory pain after different magnet pole applications is presented in **c**. *represents p < 0.05 versus the Keto group, ^†^represents p < 0.05 versus the SPIO (N-N) group, ^‡^represents p < 0.05 versus the SPIO (N-S) group, ^§^represents p < 0.05 versus the Keto-SPIO (N-N) group, |p < 0.05, compared to the Keto-SPIO group; Values are mean ± S.E.M

The optimal intrathecal injection dose of the ketorolac and SPIOs was determined based on the dose-related analgesic findings in the Fig. 4a, b (n = 2–5 per group). The increases in analgesic efficacy as affected by the magnetic field guidance mode and chemical conjugation are presented in Fig. 4c (n = 5–6 per group). The data demonstrate that both magnetic field orientation groups (the N-S or N-N group) provided better analgesia than the other two groups (ketorolac or SPIO alone) without applying a magnetic field (Ketorolac-SPIO no magnetic field). And the N-S group showed even better efficacy than the N-N group. Interestingly, SPIO alone also provided a certain level of analgesic efficacy with or without magnetic field guidance while the magnet delivered better results (Fig 4a, c).

**Fig. 5:**
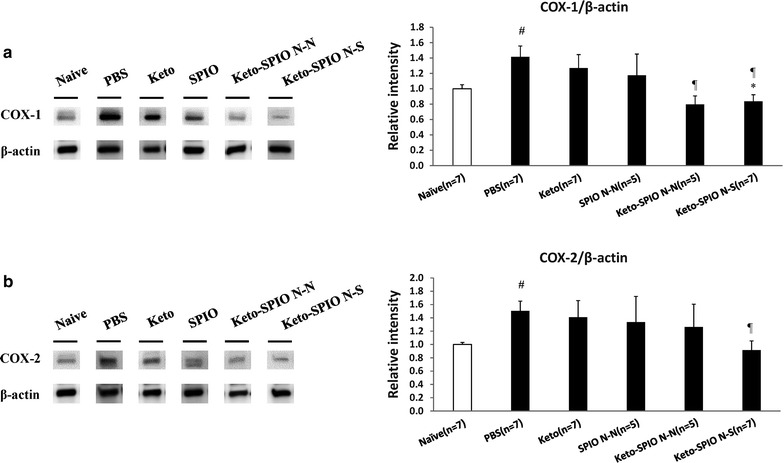
The suppression of COX-1 (**a**) and COX-2 (**b**) over the spinal cord after Keto-SPIO administration. *represents p < 0.05 versus the Keto group, ^¶^represents p < 0.05 versus the PBS sham group, ^#^represents p < 0.05 versus the Naïve group. Values are mean ± S.E.M

COX protein expression of the lumbar spinal tissues (left lesion sensory quarter only) harvested after the experiment was revealed using Western blot. Both the COX-1 and COX-2 protein expressions were significantly suppressed after the Keto-SPIO treatment (Naïve, PBS, Keto, SPIO, Keto-SPIO (N-N), Keto-SPIO (N-S): COX-1: 1.00 ± 0.05, 1.42 ± 0.14, 1.27 ± 0.18, 1.17 ± 0.28, 0.80 ± 0.11 (*p *< 0.01 vs. PBS), 0.84 ± 0.09 (*p *< 0.01 vs. PBS; *p *= 0.047 vs. Keto), COX-2: 1.00 ± 0.03, 1.50 ± 0.15, 1.41 ± 0.25, 1.34 ± 0.39, 1.26 ± 0.34, 0.91 ± 0.37 (*p *= 0.013 vs. PBS) (see Fig. 5, n = 5 ~ 7 per group; raw data are presented in the Additional file 1: Figure S1a, b; Although COX-1 and COX-2 expressed a wide variation, ketorolac still exhibited its suppression effect on COX-1 and COX-2 with or without the conjugated nanoparticles).

## Discussion

Magnet field guided tumor targeting therapy using SPIO nanoparticles have been reported in animal as well as human studies since 1981 and 1996, respectively [12, 13]. However, their clinical applications are yet to be further developed. Recently, recombinant tissue plasminogen activator conjugated magnetic nanocarriers were demonstrated for targeted thrombolysis in a rat model [14]. Moreover, magnetic nanogel with entrapped ropivacaine also was demonstrated to significantly improve analgesic efficacy as evidenced by the increase in paw withdraw latencies [15]. In this study, we are the first report using polarity modulated magnetic field to guide and retain a clinically available pain control medication carried by SPIO NPs at the target spinal cord lesion site thus promote analgesia. The observation that the SPIO NPs alone could derive a specific level of analgesia was consistent with our previous report (Fig. 4a) [6]. Comparatively, augmented analgesia effects were obtained when SPIO NPs were conjugated with NSAID for intrathecal administration plus polarity modulated magnetic field guidance.

One of the important concerns in nanomedicine is the effective loading, transportation, delivery and release of the payload medicine to the desired target. In this study, ketorolac was covalently conjugated onto SPIO NPs through formation of an amino-bond between the carboxyl group on the ketorolac structure and the amine group on the nanoparticle surface. Although a covalent bond provides very stable conjugation that protects the payload chemicals from premature release before arriving at their final destination, determining a method by which to release these chemicals at a target site and thus allow them to exert their functions can be a challenge. From the significant suppression of COX expression demonstrated in the Western blot, the pharmaceutical activity of ketorolac was not only well preserved but also further augmented by the SPIO plus magnetic field guidance. Ketorolac is a first generation NSAIDSs developed in 1989 that acts through blocking the COX function in prostaglandin synthesis with relatively higher affinity than that of COX1 and a fast dissociation rate (*K*_*off*_ at 0.1 S^−1^) [16]. A recent study reported the synthesis of ketorolac-ester as a potential prodrug for transdermal delivery. The prodrug was accelerated for transdermal delivery and rapidly metabolized to ketorolac in the skin and the plasma following a first-order kinetics [17]. These observations suggest the possibility that the Keto-SPIO NPs may have been converted into free Ketorolac and SPIO NPs in the CSF where both components act synergistically to create analgesic efficacy. In addition, the SPIO NPs acting as both pharmaceutical entities and nanocarriers also play a critical role in the augmentation of local drug concentration through magnetic field modulated local trapping. Such a local enrichment design is very important for Ketorolac, not only in regard to retaining an adequate pharmaceutical local dose for this short-acting yet potent analgesic, but also to reduce its well-known systemic side effects through spatial confinement of its pharmacodynamic distribution. Due to its notorious side effects, Ketorolac is usually not prescribed for more than 5 days despite its advantage of a strong analgesic effect without respiration suppression such as occurs with opioids. Despite the fact that magnetic field assisted anesthesia of nanogel trapped ropivacaine was reported recently, only the effect of magnetic field exposure duration rather than polarity alignment was explored [15]. However, Keto-SPIO conjugates may also directly act as an analgesic in spinal cord. Further investigation was needed to solve this issue.

In this study, we evaluated the influence of magnetic field polarity on the efficiency of physical force guided nanomedicine delivery. The results showed that different polarity alignments do significantly affect the analgesic effect of Ketorolac-SPIO NPs. Compared to previous reports in magnetic force guided delivery in which magnetic polarity alignment was rarely discussed, this study is among the few pilot reports to demonstrate its importance and to give practical recommendations for future clinical development [12, 13]. We propose that N-S polarity alignment will provide a field to align the SPIO NPs through the target lesion relatively homogenously along the magnetic line of force, thus enabling a longer tissue retention time for sustained in situ drug release. The N-N polarity alignment, on the other hand, may have a different magnetic line of force distribution and the real analgesia mechanism need further investigation. The IVIS^®^ noninvasive quantitative molecular imaging conducted in this study provided direct bio-distribution evidence to support the supposition that N-S magnet polarity alignment can trap more Ketorolac-SPIO NPs inside the lesion in the lumbar area of the paw of the CFA pain model animal, therefore providing superior analgesia. This hypothesis was also consistent with the observed COX protein suppression (Fig. 5a, b). However, the detailed in situ releasing kinetics of the Ketorolac-SPIO NPs and the derived analgesic effects require further investigation.

The potential neurotoxicity of SPIO NPs in the spinal cord is also considered. In the analgesia measurement period, except analgesia behavior, we also noticed that there were no observable behavioral changes (like bizarre walk, tremor, exciting up and down or tail swinging) in mice during 3–4 h observation period. Furthermore, in the following 1 week observation period in animal cage, there is no body weight difference for keto-SPIO injected animals or SPIO NPs alone groups compared with other animals which suggest the possible good biocompatibility for acute and sub-acute phase. This observation is also consistent with previous biocompatibility evaluations of SPIO NPs [12, 18, 19]. However, the long-term toxicity and pharmacokinetics in the spinal cord, certainly require further evaluation.

## Conclusions

We demonstrated a novel unique magnetic force guided local analgesic delivery using a clinically available NSAID through covalent conjugation to SPIO NPs. This integration provided outstanding analgesic effects through delicate N-S trans-lesional magnet polarity alignment. The integrated non-contact physical force guided nanomedicine delivery and the synergy of analgesic efficacy of both SPIO NPs per se. The small molecular compound Ketorolac may inspire future analgesic approach designs while reducing the side effects of NSAIDs.

## Additional file

**Additional file 1: Figure S1.** Blotting raw spinal cord data after ketorolac administration.
