# Supplementary figures and images for: Magnetic field distribution modulation of intrathecal delivered ketorolac iron-oxide nanoparticle conjugates produce excellent analgesia for chronic inflammatory pain
Source: J Nanobiotechnology. 2018 May 16;16:49. doi: 10.1186/s12951-018-0375-9 (PMC5956965; doi:10.1186/s12951-018-0375-9)

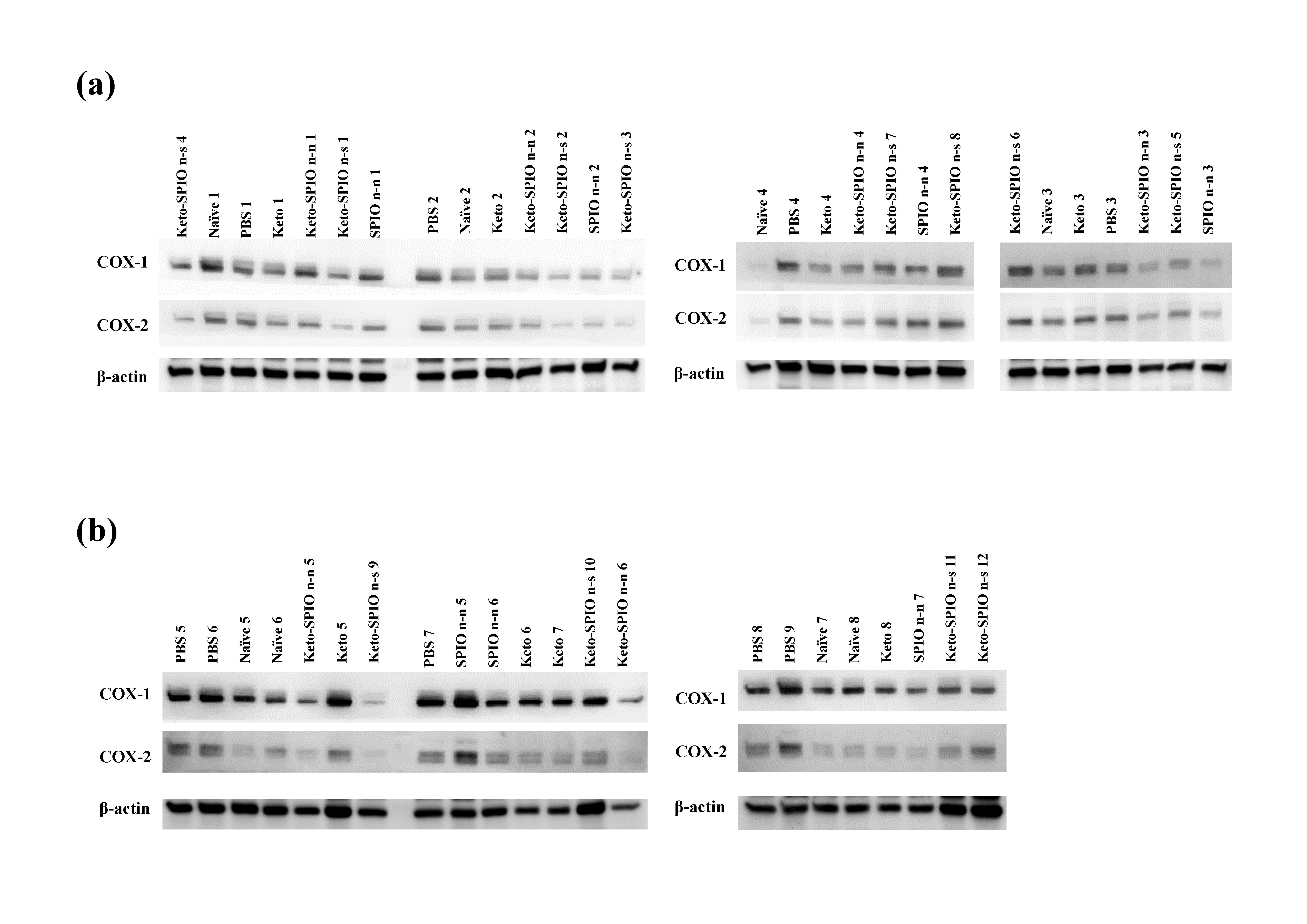

Supplement: Supplementary file 1 — Additional file 1: Figure S1. Blotting raw spinal cord data after ketorolac administration. [file 12951_2018_375_MOESM1_ESM.tiff]
